# Supplementary material for: Magnetostructural Properties of Some Doubly-Bridged Phenoxido Copper(II) Complexes
Source: Molecules. 2023 Mar 14;28(6):2648. doi: 10.3390/molecules28062648 (PMC10058975; doi:10.3390/molecules28062648)
Supplement: Supplementary file 1 [file molecules-28-02648-s001.zip › molecules-2248135-supplementary.pdf]

SUPPLEMENTARY MATERIALS

Magnetostructural Properties of Some Doubly-Bridged Phenoxido Copper(II) Complexes

Salah S. Massoud [1,2,*], Febee R. Louka [1], Madison T. Dial [1], Nahed N. M. H. Salem [2], Roland C. Fischer [3], Ana Torvisco [3], Franz A. Mautner [4], Kai Nakashima [5], Makoto Handa [5] and Masahiro Mikuriya [6,*]

[1] *Department of Chemistry, University of Louisiana at Lafayette, P.O. Box 43700, Lafayette, LA 70504, USA*
[2] *Department of Chemistry, Faculty of Science, Alexandria University, Moharam Bey, 21511 Alexandria, Egypt*
[3] *Institut für Anorganische Chemische, Technische Universität Graz, Stremayrgasse 9/V, A-8010 Graz, Austria*
[4] *Institut für Physikalische and Theoretische Chemie, Technische Universität Graz, Stremayrgasse 9/II, A-8010, Graz, Austria*
[5] *Department of Chemistry, Interdisciplinary Graduate School of Science and Engineering, Shimane University, 1060 Nishikawatsu, Matsue 690-8504 Japan*
[6] *Department of Applied Chemistry for Environment, School of Biological and Environmental Sciences, Kwansei Gakuin University, 1 Gakuen Uegahara, Sanda 669-1330 Japan*

Contents **page #**

Figure S1. Packing view of **3** …………………………………………………… S2
Figure S2. Packing view of **4** ……………………………………………………. S2
Figure S3. Packing view of **9** ……………………………………………………. S3
Table S1. Crystallographic data and processing parameters of **3**, **4** and **9** ……………………………………………………………………….. S4
Figure S4. Temperature dependence of magnetic susceptibility and magnetic moment of **2** ………………………………………………………….. S5

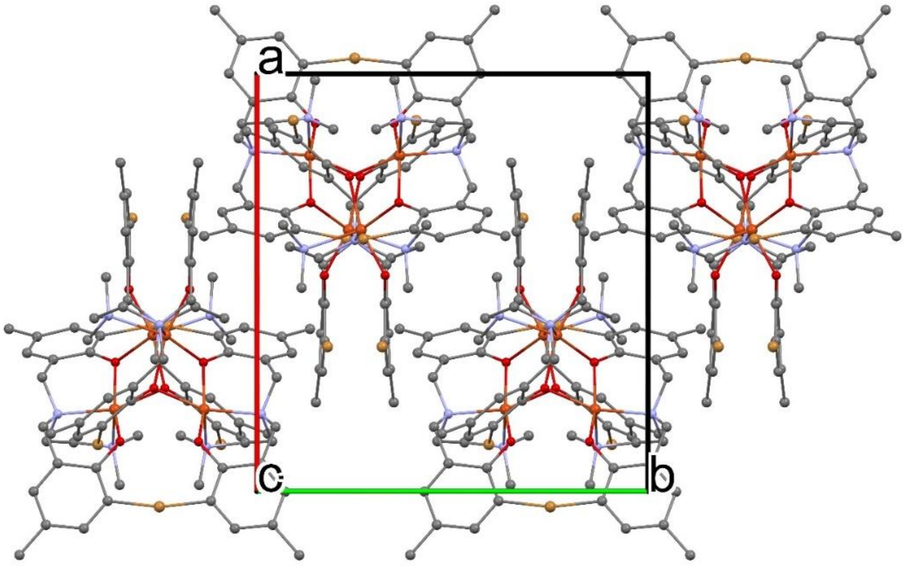

Figure S1. Packing view of **3**

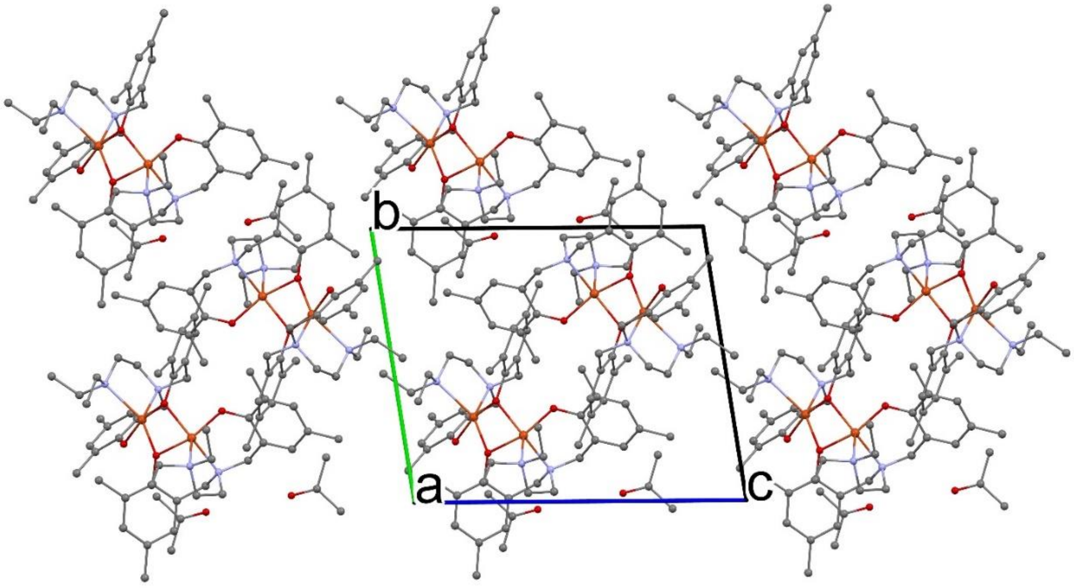

Figure S2. Packing view of **4**

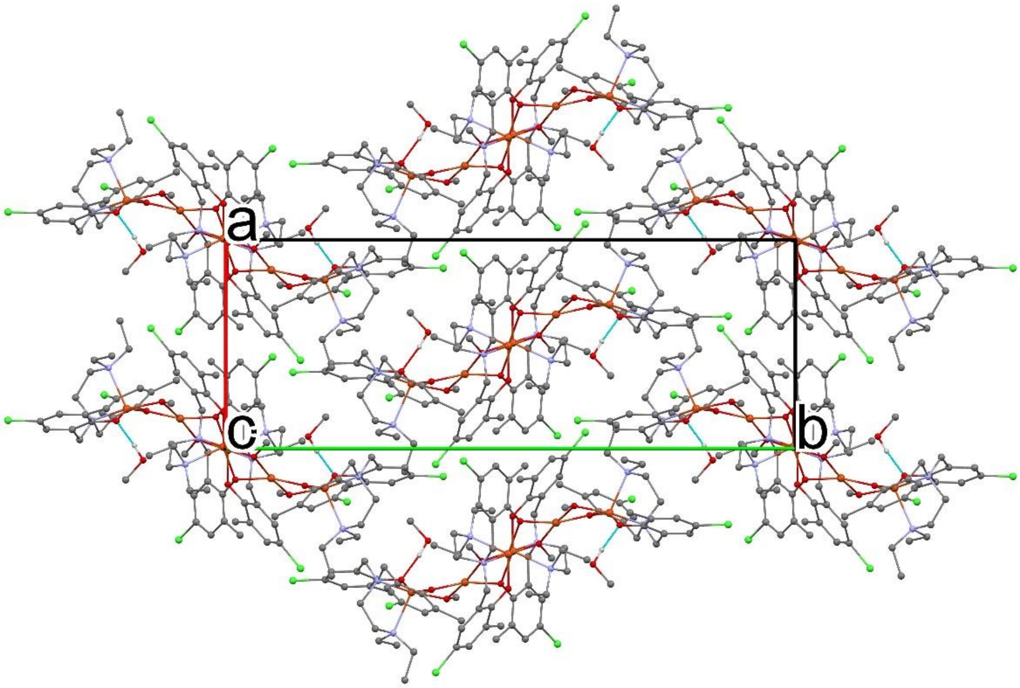

Figure S3. Packing view of **9**

Table S1. Crystallographic data and processing parameters of **3**, **4** and **9**.

Compound	**3**	**4**	**9**
Empirical formula	$C_{40}H_{48}Br_4Cu_2N_4O_4$	$C_{51}H_{74}Cu_2N_4O_5$	$C_{47}H_{62}Cl_4Cu_3N_4O_7$
Formula mass	1095.53	950.22	1131.45
System	Monoclinic	Triclinic	Monoclinic
Space group	$P2_1/c$	P-1	$P2_1/n$
a (Å)	15.433(3)	12.6538(12)	10.7647(5)
b (Å)	14.198(3)	13.0192(12)	29.4374(14)
c (Å)	20.973(4)	15.4646(15)	16.5922(8)
α (°)	90	95.347(5)	90
β (°)	100.460(7)	101.633(5)	90.673(2)
γ (°)	90	103.880(4)	90
V ($Å^3$)	4519.2(16)	2395.5(4)	5275.5(4)
Z	4	2	4
θ max (°)	26.000	30.558	24.999
Data collected	82442	133738	159804
Unique refl.	8840	14144	9251
Parameters	495	573	600
Goodness-of-Fit on F^2	1.069	1.057	1.109
R1 / wR2 (all data)	0.1102 / 0.2845	0.0528 / 0.1336	0.0357 / 0.0923

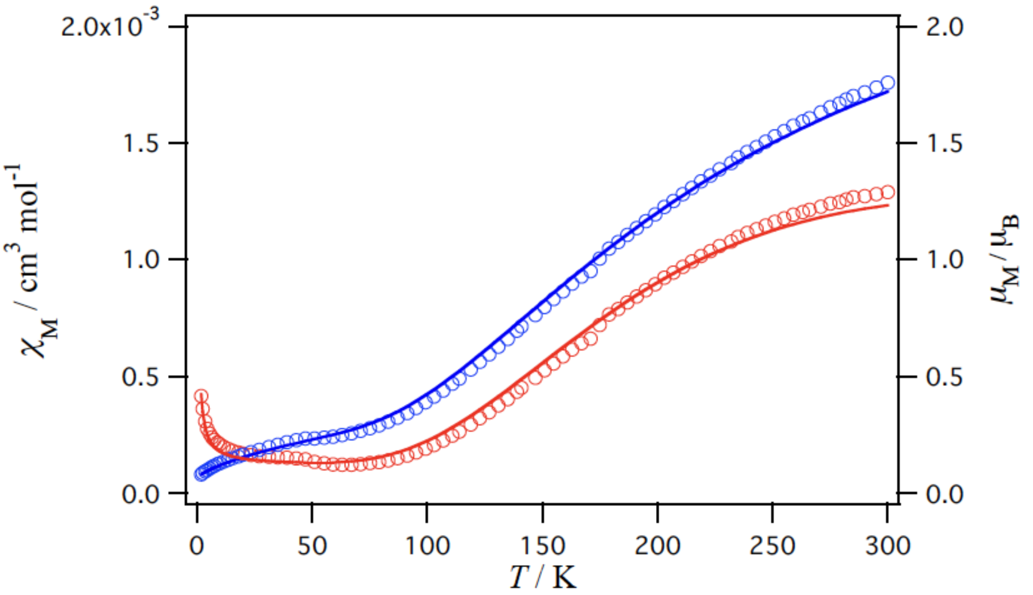

Figure S4. Temperature dependence of magnetic susceptibility χ_M (○) and magnetic moment μ_{eff} (○) of **2**.
